# A single dose of the γ-secretase inhibitor semagacestat alters the cerebrospinal fluid peptidome in humans

**DOI:** 10.1186/s13195-016-0178-x

**Published:** 2016-03-07

**Authors:** Mikko Hölttä, Robert A. Dean, Eric Siemers, Kwasi G. Mawuenyega, Wendy Sigurdson, Patrick C. May, David M. Holtzman, Erik Portelius, Henrik Zetterberg, Randall J. Bateman, Kaj Blennow, Johan Gobom

**Affiliations:** Department of Psychiatry and Neurochemistry, Clinical Neurochemistry Laboratory, Institute of Neuroscience and Physiology, The Sahlgrenska Academy, University of Gothenburg, SU/Mölndal Hospital, 431 80 Mölndal, Sweden; Lilly Research Laboratories, Eli Lilly and Company, Lilly Corporate Headquarters, Indianapolis, IN 46285 USA; Department of Neurology, Washington University School of Medicine, 660 South Euclid, Campus Box 8111, St. Louis, MO 63110 USA; Alzheimer’s Disease Research Center, Washington University School of Medicine, 660 South Euclid, Campus Box 8111, St. Louis, MO 63110 USA; Hope Center for Neurological Disorders, Washington University School of Medicine, 660 South Euclid, Campus Box 8111, St. Louis, MO 63110 USA; UCL Institute of Neurology, Queen Square, London, WC1N 3BG UK

## Abstract

**Background:**

In Alzheimer’s disease, beta-amyloid peptides in the brain aggregate into toxic oligomers and plaques, a process which is associated with neuronal degeneration, memory loss, and cognitive decline. One therapeutic strategy is to decrease the production of potentially toxic beta-amyloid species by the use of inhibitors or modulators of the enzymes that produce beta-amyloid from amyloid precursor protein (APP). The failures of several such drug candidates by lack of effect or undesired side-effects underscore the importance to monitor the drug effects in the brain on a molecular level. Here we evaluate if peptidomic analysis in cerebrospinal fluid (CSF) can be used for this purpose.

**Methods:**

Fifteen human healthy volunteers, divided into three groups, received a single dose of placebo or either 140 mg or 280 mg of the γ-secretase inhibitor semagacestat (LY450139). Endogenous peptides in CSF, sampled prior to administration of the drug and at six subsequent time points, were analyzed by liquid chromatography coupled to mass spectrometry, using isobaric labeling based on the tandem mass tag approach for relative quantification.

**Results:**

Out of 302 reproducibly detected peptides, 11 were affected by the treatment. Among these, one was derived from APP and one from amyloid precursor-like protein 1. Nine peptides were derived from proteins that may not be γ-secretase substrates *per se*, but that are regulated in a γ-secretase-dependent manner.

**Conclusions:**

These results indicate that a CSF peptidomic approach may be a valuable tool both to verify target engagement and to identify other pharmacodynamic effects of the drug. Data are available via ProteomeXchange with identifier PXD003075.

**Trial registration:**

NCT00765115, registered 30/09/2008.

**Electronic supplementary material:**

The online version of this article (doi:10.1186/s13195-016-0178-x) contains supplementary material, which is available to authorized users.

## Background

Alzheimer’s disease (AD) is the most common form of dementia, affecting an increasing number of people worldwide and presenting a severe social and economic burden. A central aspect of the AD pathology is the formation of plaques in the brain, consisting of aggregated amyloid-β (Aβ) peptides [1]. A variety of Aβ peptides are produced by enzymatic cleavage of the amyloid precursor protein (APP) by β-secretase (BACE) and γ-secretase, of which the 42 amino acid long Aβ peptide (Aβ_42_) is most prone to aggregation [2] and has been proposed to be the driving force in the disease [3, 4]. Several treatment strategies currently under investigation target the production or clearance of Aβ, e.g., immunotherapy with anti-Aβ antibodies, and treatment with γ-secretase inhibitors (GSIs) and BACE inhibitors [5, 6]. While some of these strategies have yielded positive results in preclinical studies, they have so far been unsuccessful in slowing cognitive decline in human AD subjects [7]. The GSI semagacestat showed promising effects in animal models [8] and also decreased the amount of newly produced Aβ in humans [9], but a phase III clinical trial failed to reach clinical endpoints and the drug turned out to have clinically significant adverse cognitive effects [10, 11].

Monitoring drug treatment effects on a molecular level is important to determine if a drug affects the intended target protein, but also to examine if other proteins are affected. The aim of this study was to test if an unbiased peptidomic approach can be used for this purpose. Previous studies have demonstrated the presence of a large number of endogenous peptides in cerebrospinal fluid (CSF) [12–16]. While the majority of CSF proteomic studies to date follow the strategy of analyzing proteins digested with trypsin, endogenous peptides – the CSF peptidome – may also be a valuable source of biomarkers, particularly for studying biological events involving proteolytic processing. Recently, we reported on a method for multiplex quantitative peptidomic analysis in CSF, based on isobaric labeling using the tandem mass tag (TMT) approach [17] combined with liquid chromatography-mass spectrometry (LC-MS) [18].

In the current study we aim, by analyzing CSF from healthy subjects treated with the GSI semagacestat, to test if this approach can be used to detect drug treatment effects on proteolytic processing in the brain, to identify substrates affected by the drug and to quantify those effects. This is, to our knowledge, the first study that explores an endopeptidomic approach for the discovery of pharmacodynamic biomarkers in human CSF.

## Methods

### Experimental design and statistical rationale

CSF from a previous study was used, in which 20 healthy human volunteers received either placebo or 100 mg, 140 mg, or 280 mg semagacestat, and CSF was sampled every hour via an indwelling catheter in the lumbar thecal sac for 36 h following oral drug administration [9, 19] (https://clinicaltrials.gov/ct2/show/NCT00765115). The study was approved by the Washington University Human Studies Committee, and was performed in compliance with the Helsinki Declaration. All participants and caregivers gave written informed consent. In the current study, CSF from the placebo group, the 140 mg group and the 280 mg group, sampled at 0 h, 3 h, 9 h, 12 h, 18 h, and 36 h were used (Table 1). The time points were selected based on the results in previous studies [9, 19], where a maximum Aβ inhibitory effect was seen between 9 and 12 h, and the values returned to approximately the same as for the placebo group at 36 h.

**Table 1:** Distribution of the CSF samples within the TMT 6-plex sets

TMT label	126	127	128	129	130	131
Time point	0 h	3 h	9 h	12 h	18 h	36 h

The CSF samples were labeled with TMT 6-plex reagents according to the table, and the ratios for the time points were calculated against the 0 h value. *CSF* cerebrospinal fluid, *TMT* tandem mass tag

**Fig. 1:**
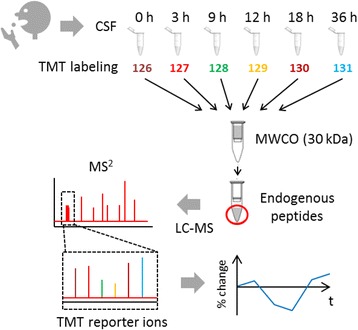
Study design. CSF sampled at several time points following oral administration of semagacestat was subjected to labeling using TMT reagents 128-131. The combined labeled samples from each participant (TMT 6-plex set) were centrifuged through a 30 kDa molecular weight cut-off filter. The flow-through, containing the endogenous peptide fraction, was analyzed by LC-MS. The relative change in concentration of each identified peptide was calculated from the TMT reporter ion signals. *CSF* cerebrospinal fluid, *TMT* tandem mass tag, *LC-MS* liquid chromatography-mass spectrometry

The experimental design is shown in Fig. 1. Neat CSF sampled at six consecutive time points before and after drug administration was labeled with isobaric TMT reagents, using a protocol recently developed in our laboratory [18]. Briefly, 100 μl aliquots of neat CSF from each participant and time point was subjected to reduction and carbamidomethylation of cysteines followed by isobaric labelling using TMT 6-plex amino-reactive reagents (Thermo Scientific). The CSF sample corresponding to time point zero from each participant was labeled with TMT-126, the next time point from the same participant with TMT-127, and so on (Table 1). The six samples from each participant were then combined into one TMT 6-plex set. The CSF samples from one participant were then combined into one TMT6-plex set. The TMT6-plex sets were subjected to ultrafiltration using 30 kDa molecular weight cut-off filters (Vivacon 2 HY, Sartorius Stedim). The flow-through, containing the endogenous peptide fraction, was desalted on C_18_ cartridges (SEP-PAK, Waters), lyophilized and stored at -80 °C pending analysis. The CSF samples were analyzed by LC-MS in two technical replicates to improve the identification and quantification overlap between study participants.

### Liquid chromatography-mass spectrometry

Endogenous peptides were reconstituted in 12 μl of 2 % acetonitrile, 0.1 % trifluoroacetic acid. A sample of 6 μl was analyzed with nano-LC (Ultimate 3000, Thermo Scientific) with a C_18_ trap column, and a C_18_ separation column, coupled to a Q-Exactive electrospray ionization mass spectrometer (Thermo Scientific). The LC mobile phases were A: 0.05 % formic acid, and B: 84 % acetonitrile, 0.05 % formic acid. The samples were separated with a 160 minute gradient running from 3 % mobile phase B to 45 % mobile phase B. The mass spectrometer was operated in the positive ion mode. The instrument settings for the MS scans were: resolution 70,000; *m/z* range 400-1600; max injection time 250 ms; AGC target 1e6. Data-dependent acquisition was used to record up to 10 consecutive fragment ion spectra (MS2) per full scan spectrum, selecting precursor ions in decreasing order of intensity, and using 20 s dynamic exclusion, and charge state exclusion to exclude signals with unassigned charge, charge 1 and >5. The isolation window was set to 1.2 *m/z*. The instrument settings for the MS2 scans were: resolution 35,000 for endogenous peptides and 17,500 for tryptic peptides; fixed first mass *m/z* 100; max injection time 120 ms for endogenous peptides and 60 ms for tryptic peptides; AGC target 1e5. Blank injections of mobile phase B were performed between the samples to avoid carry-over.

### Data analysis

The data from the two replicate runs of each participant were used in a MudPIT search using the software Proteome Discoverer 1.4 (Thermo Scientific). Protein identification was performed using Mascot v. 2.3 (Matrix Sciences, UK), searching the human subset of the UniProtKB/Swiss-Prot database (Release 2013-10, 88,266 sequences). The endogenous peptides were searched with the following settings: fixed modifications: TMT 6-plex modification of peptide N-termini and lysines, and carbamidomethylation of cysteines; variable modification: oxidation of methionine. The mass error tolerance was set to 10 ppm in MS mode and 20 milli-mass units in MS/MS mode. The peptide cut-off score for individual spectra was 10. A target false discovery rate value of 5 %, based on decoy database searches, was used as identification criterion. The quantification was done on the MS/MS level where reporter ions from the TMT 6-plex reagents were used for relative quantification. The mass spectrometry proteomics data have been deposited to the ProteomeXchange Consortium [20] via the PRIDE partner repository with the dataset identifier PXD003075 and 10.6019/PXD003075".

The CSF sample collected at time point 0 h was used as reference sample for each individual and the change in peptide abundance at later time points was calculated relative to the peptide’s abundance at this time point.

A majority of the identified endogenous peptides increased between each time point regardless of whether the participant received placebo or the active substance. This has been observed for other analytes measured in these samples [9, 19]. The average increase over 24 h was 47 %. This drift was normalized toward baseline by subtracting the median ratio of the placebo group from the two groups that received the active substance for each peptide and time point.

The data were evaluated according to the following criteria: 1) the alterations of the peptide levels should be dose dependent; 2) the levels should not fluctuate substantially between each time point; 3) spikes at isolated time points were not considered to be drug dependent; 4) values from at least four individuals in each group were required to be present; and 5) the peptide levels should return toward the placebo groups’ values at 36 h. Because peptide abundances cannot be assumed to be normally distributed, the non-parametric Friedman test in the software PASW Statistics 18 (IBM) was used to evaluate the remaining peptides, employing a significance threshold of p < 0.05.

## Results

The aim of the current study was to test if an unbiased endopeptidomic approach could be used to detect changes in the CSF endopeptidome in response to drug treatment. In total, 1798 endogenous peptides were identified and quantified in at least one TMT 6-plex set (corresponding to one individual). Of these, 302 endogenous peptides fulfilled the set quantification criteria of being identified and quantified in at least four participants in each of the three treatment groups (Additional file 1: Table S1). Of these, 11 peptides were significantly affected (p < 0.05) by treatment with semagacestat (Table 2).

**Table 2:** Peptides affected by drug treatment

Peptide	Protein	p		Time at maximum decrease (h)	% maximum decrease
		140 mg	280 mg		
EDVGSNK	Amyloid Precursor Protein and Amyloid beta A4 protein^ab^	0.36	0.0035	9	42
DELAPAGTGVSREAVSG	Amyloid-like protein 1^ab^	0.077	0.0018	12	32
SVQPDSPTDVNQENVPS	Tachykinin-3	0.065	0.0041	9	38
VTEDDEDEDDDKE	Testican-1	0.25	0.0039	18	26
AVTEDDEDE	Testican-1	0.065	0.0025	18	19
DDEDEDDDKE	Testican-1	0.41	0.0039	18	26
EKLPGQGVHSQGQGPGAN	Golgi apparatus protein 1^a^	0.11	0.0020	18	20
DFLAEGGGVR	Fibrinogen alpha chain	0.66	0.0024	9	36
EPPPPPEPA	CD99 antigen-like protein 2^a^	0.69	0.0041	18	24
TVVQPSVGAAAGPVVPPCPGRIRHFKV	Alpha-2-HS-glycoprotein	0.86	0.0039	9	27
DPNCSCATGGSCTCAGSCKCKE	Metallothionein-1E	-	0.0050	12	42

Endogenous CSF peptides that decreased in abundance after treatment with semagacestat

*CSF* cerebrospinal fluid

^a^transmembrane protein

^b^previously reported gamma secretase substrate

**Fig. 2:**
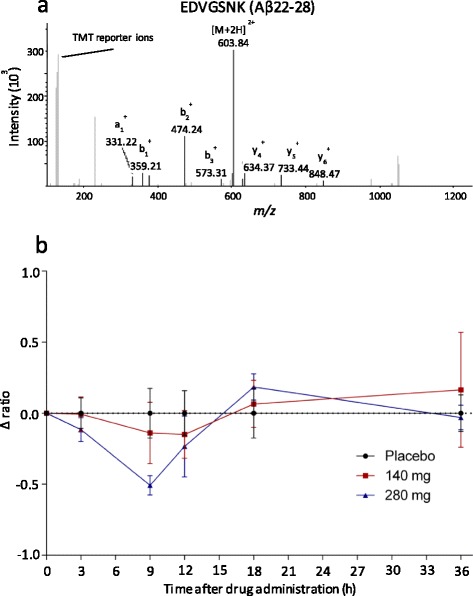
β-amyloid 22-28. The peptide EDVGSNK, constituting fragment 22-28 of β-amyloid within APP. **a** annotated MS2 spectrum. **b** Relative abundance of the peptide after semagacestat treatment. In the 280 mg dosage group the concentration of the peptide decreased to a minimum of 42 % at 9 h (p = 0.0035), while in the 140 mg dosage group the minimum relative abundance was 13 % at 12 h (p = 0.36). Graphed data are medians with median absolute deviations. *APP* amyloid precursor protein

One of these was the peptide EDVGSNK, constituting fragment 22-28 of Aβ (Fig. 2). Within the SwissProt sequence database, this peptide sequence is unique to APP. As the GSI blocks production of Aβ by inhibiting c-terminal cleavage from APP, decreased EDVGSNK indicates that the drug hits the target and affects Aβ production. The abundance of the peptide decreased by a maximum of 42 % after 9 h in the group that received 280 mg of semagacestat (p = 0.0035) (Fig. 2b). A 13 % decrease was observed in the group that received 140 mg.

**Fig. 3:**
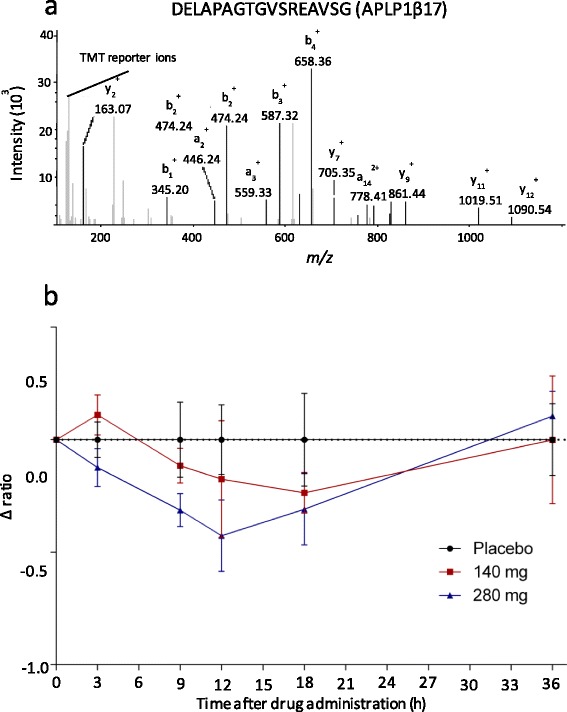
APL1β17. The peptide DELAPAGTGVSREAVSG, constituting fragment APL1β17 from amyloid-like protein 1 (APLP1). **a** Annotated MS2 spectrum. **b** Relative abundance of the peptide after semagacestat treatment. In the 280 mg dosage group the concentration of the peptide decreased to a minimum of 32 % at 12 h (p = 0.0018), and in the 140 mg group the minimum relative concentration was 15 % at 18 h (p = 0.078). Graphed data are medians with median absolute deviations

Another peptide that changed in abundance was DELAPAGTGVSREAVSG (Fig. 3). The peptide is a fragment of amyloid-like protein 1 (APLP1), also known to be cleaved by γ-secretase [21]. The peptide, denoted APLP1β17, decreased by a maximum of 32 % after 12 h in the group that received 240 mg (Fig. 3b).

Of the remaining peptides, two were derived from proteins with transmembrane regions (Golgi apparatus protein 1 and CD99 antigen-like protein 2), which are thus potential gamma γ-secretase substrates.

## Discussion

While human CSF has been found to contain a large number of endogenous peptides, relatively little research has been performed to explore the potential of this class of molecules as a source of biomarkers. The current study is, to our knowledge, the first in which an explorative endopeptidomic approach has been employed to identify drug treatment biomarkers in CSF. Of 303 endogenous peptides that could be reproducibly identified in at least 50 % of the study participants, 11 differed significantly in abundance compared to t_0_, at one or several time points, and of these, two were derived from known gamma secretase substrates, which thus would be expected to be affected by the treatment. These proportions support our hypothesis that an unbiased peptidomic approach is sufficiently specific to detect treatment-induced changes on the peptide level.

**Fig. 4:**
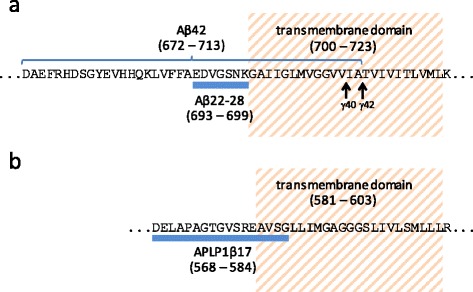
Transmembrane location of Aβ22-28 and APLP1β17. Location of the peptides (**a**) Aβ22-28 and (**b**) APLP1β17 relative to the transmembrane region of their respective protein sequences. The γ-secretase cleavage sites in APP are indicated with *arrows. APP* amyloid precursor protein

While Aβ22-28, which decreased upon semagacestat treatment (Fig. 2), has not been previously identified in CSF, previous studies reported decreased levels of Aβ_x-38_, Aβ_x-40_, and Aβ_x-42_ in CSF after a single dose [19] and a decrease in the production of total Aβ in a dose dependent fashion [9]. The γ-secretase protease complex acts within the cellular membrane and cleaves its substrate proteins within their transmembrane-spanning region. Although position 28 in the Aβ sequence is at the edge of the transmembrane region (Fig. 4a), it has not been reported as a γ-secretase cleavage site. Through mass spectrometry-based studies we have learned that several different Aβ peptides are present in the CSF and that they are affected differently by treatment [22]. For example, we have previously shown, using immunoprecipitation in combination with MS, that chronic treatment of AD participants with semagacestat results in increased levels of Aβ1-15 and Aβ1-16 [23]. Even though these short Aβ peptides are not generated directly by γ-secretase cleavage, inhibition of γ-secretase may induce an accumulation of C99, the transmembrane carboxyl-terminal domain of APP, which in turn is cleaved by α- and β-secretases resulting in increased levels of Aβ1-15 and Aβ1-16 [24]. Thus, the observed decrease in Aβ22-28 does not necessarily imply that γ-secretase cleaves at position 28 but may instead reflect decreased production of a longer Aβ peptide, which is then further cleaved by other proteases to Aβ22-28.

That full-length Aβ40 or Aβ42 was not detected is a limitation of this study, resulting from steps in the sample preparation and analytical conditions: C_18_ chromatographic media, used in LC-MS, may interact too strongly with the highly hydrophobic longer Aβ peptides, preventing their elution. Furthermore, following lyophilization, the samples were reconstituted in acidified aqueous solution, in which the solubility of the longer Aβ peptides is low. Using a complementary sample preparation, for example employing basic solvents, and C_4_ or C_8_ chromatographic media, may provide a more complete view on the processed peptides.

APL1β17, a 17-amino acid long peptide located near the transmembrane region of APLP1 (Fig. 4b), also decreased in response to the treatment (Fig. 3). The peptide showed a maximum decrease 12 h after drug intake, which matches observations of truncated Aβ peptides after treatment with semagacestat [19]. Like APP, APLP1 also is a known substrate for γ-secretase [21]. It is processed around the transmembrane region by the same enzymes as APP, resulting in the formation of Aβ-like peptides [21, 25]. Specifically, APLP1β25, APLP1β27, and APLP1β28 from APLP1 have been shown to be generated by β - and γ-secretase cleavage [25]. These peptides have, however, not been found to be a part of amyloid plaques [25], in contrast to full length APLP1 [26].

The overall levels of endogenous peptides increased over time in all groups; this has also been observed in a previous study of the same CSF samples [19]. The increase may be caused by changes in the cephalic to caudal gradient when sampling CSF repeatedly over 36 hours. Therefore, in the current study, the increase was corrected for each endogenous peptide by subtracting the median value of the placebo group from the median value of the treatment groups.

The majority of the identified peptides that were affected by semagacestat treatment were not previously known γ-secretase substrates. Notably, several of these were derived from proteins without transmembrane domains and would thus not be expected to be γ-secretase substrates. This observation may indicate a direct or indirect modulation of protein metabolism through inhibition of γ-secretase, suggesting that GSIs may have even broader biological effects than predicted from γ-secretase substrates alone. The clinical trials with the drug failed because of a number of adverse effects, most importantly cognitive worsening, that occurred for unknown reasons. The changes observed in many of the endogenous peptides may not necessarily be the direct result of γ-secretase activity, but may be caused by downstream γ-secretase dependent processing or could be the result of drug effects on other proteases. These changes demonstrate the importance of analyzing the global composition of the samples in similar studies to elucidate whether a drug affects processes other than those expected or gives rise to unintended alterations among endogenous peptides or proteins. The information obtained in these experiments might also identify novel candidates for the enzymes whose functions are being modified with a treatment *in vivo*, which otherwise can be hard to identify/detect.

## Conclusions

Among the 303 endogenous peptides identified in total, a significant change in abundance in response to GSI treatment was detected in 11 peptides, and among those, two were derived from proteins that are known substrates to γ-secretase. These results support our hypothesis that an unbiased endopeptidomic analytical approach can be used to detect peptides that are affected by drug treatment, and suggest that this approach may be valuable to include in future clinical trials.

## Additional file

Additional file 1: Table S1. Identified peptides. Peptides identified and quantified in at least four participants in each of the three treatment groups. Quantitative data are presented as percent change relative to CSF sampled at the time point when the drug was administered. The values are normalized to the average relative peptide abundance in the placebo group for each time point. (XLSX 86 kb)

## Abbreviations

AD: Alzheimer’s disease
APLP1: Amyloid-like protein 1
APP: Amyloid precursor protein
Aβ: beta-amyloid
CSF: Cerebrospinal fluid
GSI: γ-secretase inhibitor
LC-MS: Liquid chromatography-mass spectrometry
TMT: Tandem mass tag
